# Dietary Tea Polyphenols Improve Meat Quality of Finishing Pigs by Enhancing Antioxidant Enzyme Activities and Modulating Gut Microbiota

**DOI:** 10.3390/ani16193135

**Published:** 2026-10-07

**Authors:** Zhixin Yu, Luhao Tian, Yanzhe Zhang, Haoneng Guo, Zhixuan Zhang, Lexuan Shi, Zengzhuang Yuan, Zeqing Lu, Yizhen Wang, Yuanzhi Cheng

**Affiliations:** 1 Zhejiang Key Laboratory of Nutrition and Breeding for High-Quality Animal Products, Zhejiang University, Hangzhou 310058, China; 12517064@zju.edu.cn (Z.Y.); 22517017@zju.edu.cn (L.T.); 22317020@zju.edu.cn (Y.Z.); 12517062@zju.edu.cn (H.G.); 22417014@zju.edu.cn (Z.Z.); 12417034@zju.edu.cn (L.S.); zqlu2012@zju.edu.cn (Z.L.); yzwang321@zju.edu.cn (Y.W.); 2 Key Laboratory of Molecular Animal Nutrition, Ministry of Education, Hangzhou 310058, China; 3 Key Laboratory of Animal Nutrition and Feed Science (Eastern of China), Ministry of Agriculture and Rural Affairs, Hangzhou 310058, China; 4 Bureau of Animal Husbandry and Veterinary, Jiaxing 314050, China; yuanzhengzhuang@126.com

**Keywords:** tea polyphenols, finishing pigs, meat quality, antioxidant capacity, colonic microbiota

## Abstract

This study aimed to investigate the effects of dietary tea polyphenols (TPs) supplementation on growth performance and meat quality. A total of 120 pigs ([Yorkshire × Landrace] × Duroc) aged 150 days with similar body weights (74.29 ± 0.55 kg), comprising equal numbers of females and castrated males, were randomly assigned to two treatments, each with three pens of 20 pigs: The Control group received a basal diet, and the TP group received the basal diet supplemented with 2000 mg/kg tea extract containing 15% TP. A 7-day adaptation preceded the 60-day feeding period. At the end of the experiment, the final bodyweight of the pigs was measured and six pigs per treatment were selected for sampling. The results showed that: (1) growth performance did not differ significantly, although feed-to-gain ratio and average daily feed intake were numerically lower in the TP group; (2) carcass weight, backfat thickness, and muscle alanine increased, whereas *longissimus dorsi* lightness and yellowness decreased (*p* < 0.05); (3) serum total superoxide dismutase and hepatic catalase and glutathione peroxidase activities increased (*p* < 0.05); (4) Chao1 richness and Shannon diversity were unchanged, and genus-level PCoA showed partial but non-significant separation of the colonic microbial communities (R = 0.1685, *p* = 0.090). Nevertheless, TP supplementation selectively altered specific bacterial taxa, with lower relative abundances of *Pseudomonadota*, *Escherichia–Shigella*, and *Ligilactobacillus* and higher relative abundances of *Solobacterium*, *norank_f__p-2534-18B5_gut_group*, and *[Ruminococcus]_gauvreauii_group*; and (5) *Catenisphaera* was positively correlated with serum total superoxide dismutase activity (*p* < 0.01), whereas the TP-enriched *norank_f__p-2534-18B5_gut_group* was positively correlated with muscle alanine content (*p* < 0.05). This study demonstrates concurrent antioxidant, meat-quality, and microbial responses to a TP-containing diet, supporting mechanistic research on a potential gut–liver–muscle association and refinement of supplementation strategies that balance antioxidant benefits with carcass fat deposition.

## Simple Summary

This research investigated whether dietary supplementation with tea polyphenols affects finishing pigs. The results indicated that pigs receiving tea polyphenols showed numerically lower feed-to-gain ratio and average daily feed intake. Supplementation increased carcass weight, backfat thickness, and alanine concentration in muscle. Tea polyphenols also enhanced serum total superoxide dismutase activity and hepatic catalase and glutathione peroxidase activities. In the colon, tea polyphenols selectively altered specific bacterial groups. The relative abundances of *Pseudomonadota*, *Escherichia–Shigella*, and *Ligilactobacillus* decreased, whereas *Solobacterium*, *norank_f__p-2534-18B5_gut_group*, and *[Ruminococcus]_gauvreauii_group* increased. *Catenisphaera* was positively associated with serum total superoxide dismutase activity, while the TP-enriched *norank_f__p-2534-18B5_gut_group* was positively associated with muscle alanine content. Overall, dietary tea polyphenols may enhance antioxidant defense, modify selected carcass and meat-quality characteristics, and reshape the colonic microbiota of finishing pigs.

## 1. Introduction

Tea polyphenols (TPs) are bioactive tea constituents investigated as feed additives across livestock and poultry production to support antioxidant defenses, immune function, and intestinal health [1,2,3]. In pig production, dietary TP promoted T-cell responses and moderated inflammatory changes in weaned piglets challenged with oxidative stress [1]. In gestating sows, supplementation with 200 or 300 mg/kg catechins increased the numbers of live-born and healthy piglets and reduced stillbirths [2]. In finishing pigs, tea powder and tea-derived supplements have also been evaluated for effects on pork composition and meat quality [4,5]. These applications provide a basis for examining TP-containing diets in relation to both physiological function and product quality.

Several controlled studies illustrate the range of responses to dietary tea constituents. Yan et al. [4] supplemented diets of Tibetan pig × Bama miniature pigs with 1%, 2%, 4%, or 6% tea powder for 60 days. Growth performance was unchanged, whereas the 2% treatment reduced muscle lightness and yellowness and the 1–4% treatments increased total and essential amino acids. Oanh et al. [5] fed finishing crossbred pigs 8, 16, or 24 g/kg green tea by-products for 10 weeks and reported reduced backfat thickness with increasing inclusion, together with changes in pork cholesterol and omega-3 fatty acids. Ding et al. [6] tested fermented tea residue at 10%, 15%, and 20% of the diet; the 15% and 20% treatments increased serum glutathione peroxidase activity and total antioxidant capacity. In contrast, Augustin et al. [7] supplied growing pigs with 10 or 100 mg green tea polyphenols/kg body weight daily for five weeks and found no effect on plasma antioxidant capacity or the measured meat-quality traits. These studies support the biological relevance of tea constituents while showing that responses vary across supplementation regimes. Tea powder, by-products, and fermented residue contain additional nutrients and bioactive compounds, so their inclusion rates require separate consideration when comparing them with tea extracts.

The specificity of the antioxidant response is particularly relevant. In weaned piglets, Wang et al. [8] found that 1 g/kg tea residue increased plasma glutathione content without significant changes in the measured antioxidant enzyme activities. Together with the finishing-pig evidence, this suggests that serum and tissue antioxidant measurements can provide complementary information. Evaluating these responses alongside muscle composition may help identify which aspects of the finishing-pig phenotype are most responsive to dietary TP.

Intestinal microbial responses offer a further connection between tea constituents and host metabolism. TP supplementation has been linked to lipid deposition and gut microbial changes in research involving Ningxiang pigs [9]. Experimental work with intestinal communities also demonstrates microbial transformation of tea theaflavins and catechins [10,11,12]. Hara et al. [13] found that dietary TP altered fecal bacterial populations and fermentation products in young pigs, including increased lactobacilli and reduced ammonia during supplementation. In Xiangling finishing pigs, tea residue combined with complex enzymes altered selected antioxidant, meat-quality, and microbial measurements [14]. Pig studies also show that host genetic background and dietary composition influence microbial community structure and function [15,16,17]. Mutual fecal microbiota transplantation between Ningxiang and Duroc × Landrace × Yorkshire pigs, followed by a strain-gavage experiment in pigs, linked microbial changes to muscle fatty acid deposition [18]. Dietary butyrate also altered muscle fiber composition and mitochondrial biogenesis in finishing pigs [19]. These pig-based findings support evaluating microbial and host responses together, while interventions involving tea residue, probiotics, or butyrate should not be treated as equivalent to TP supplementation. Studies of microbial colonization and muscle metabolism in pigs further provide a biological basis for investigating microbial–muscle relationships [20,21,22,23]. The remaining question is how colonic community changes accompany serum and hepatic antioxidant responses and muscle amino acid composition under the same TP feeding regime.

Although TP have been widely studied, their integrated effects on production traits, antioxidant status, muscle composition, and colonic microbiota in finishing pigs remain unclear. Therefore, this study aimed to evaluate the effects of dietary TP-containing tea extract supplementation on growth performance, carcass and meat-quality traits, serum and hepatic antioxidant indices, muscle amino acid composition, and colonic microbiota, and examined associations between microbial changes and host traits. These findings may support further evaluation of TP-containing tea extract as a phytogenic feed additive in finishing pigs.

## 2. Materials and Methods

### 2.1. Animals and Experimental Design

A total of 120 finishing pigs ([Yorkshire × Landrace] × Duroc), aged 150 days with similar body weights (74.29 ± 0.55 kg) and comprising 60 females and 60 castrated males, were randomly assigned to two dietary treatments with comparable initial body weights. Each treatment comprised three replicate pens of 20 pigs. The experiment was conducted at the Pig Experiment Research Farm, Zhejiang Tianpeng Group Co., Ltd. (Taizhou, China), from February to April 2025. The basal diets contained corn, soybean meal, barley, wheat bran, soybean oil, soybean hulls, lysine, and a vitamin–mineral premix (Table 1) and followed the guidance of nutrient requirements by the National Research Council (NRC, 2012) [24]. The TP diet was supplemented with 2000 mg/kg tea extract containing 15% tea polyphenols, corresponding to a TP supply of 300 mg/kg complete feed. The tea extract used in this study was derived from black tea produced from *Camellia sinensis*. Both groups otherwise received the corresponding basal diet. Pigs were housed in an environmentally controlled building in pens measuring 4.5 × 8.5 m with partially slatted concrete floors. Temperature was maintained at 15–20 °C and relative humidity at 50–65%. Each pen was equipped with an automatic feeder and nipple drinker, allowing ad libitum access to feed and water. Feed was supplied at 08:00 and 16:00 each day. The daily feed allowance was adjusted according to pen body weight, and remaining feed was weighed before each delivery. Pigs were vaccinated according to the routine immunization program used for commercial finishing pigs, including vaccines against foot-and-mouth disease (FMD), classical swine fever (CSF), porcine circovirus type 2 (PCV2), and pseudorabies. Health status was monitored through daily herd observation, environmental management, and laboratory testing. Pigs were inspected twice daily for feed intake, general behavior, respiratory signs, and fecal condition. Housing temperature and ammonia concentrations were also monitored, and strict disinfection and all-in/all-out management procedures were implemented. In addition, serum antibody levels against FMD, CSF, pseudorabies, and PCV2 were monitored periodically. Pathogen-specific PCR testing was performed when necessary, including surveillance for African swine fever (ASF). The experiment comprised a 7-day adaptation period followed by a 60-day feeding period.

### 2.2. Growth Measurements and Sample Collection

Initial and final body weights were recorded to determine average daily gain (ADG). Pigs were weighed using a floor scale at the end of the trial. Average daily feed intake (ADFI) and the feed-to-gain ratio were calculated from feed and body-weight records over the feeding period. No pigs were removed from the trial, and all pigs were included in the growth-performance assessment. At the end of the trial, two pigs whose body weights were closest to the mean body weight of their respective pen were selected from each pen for slaughter sampling, after which carcasses were weighed. Liver, *longissimus dorsi* (LD) muscle, and colonic contents were collected. Liver samples and colonic contents were transported to the laboratory under liquid-nitrogen conditions and stored at −80 °C. Colonic contents were used for 16S rRNA gene profiling, and serum and liver samples were used for antioxidant assays.

Approximately 5 mL of blood was collected from the jugular vein of each sampled pig into serum collection tubes with clot activator (KWS, Shijiazhuang, Hebei, China) and centrifuged at 3000× *g* for 10 min at 4 °C. The upper serum fraction was collected, transported to the laboratory under liquid-nitrogen conditions, and stored at −80 °C for antioxidant assays. Backfat thickness was measured using a vernier caliper (Meinaite, Shanghai, China) at the first rib, last rib, and lumbar region; the mean of these three measurements was calculated for each pig. LD muscle samples were collected at the 10th rib and maintained at 4 °C for determination of shear force, pH, meat color, drip loss, and amino acid composition.

Meat-quality measurements included drip loss, pH, marbling score, meat color score, instrumental lightness (L*), redness (a*), yellowness (b*), myoglobin, shear force, moisture, inosine monophosphate (IMP), and inosine. Instrumental color and pH were measured at the same site of the LD 24 h after slaughter. Color was measured in triplicate using a ColorFlex colorimeter (HunterLab, Reston, VA, USA), and the readings were averaged for each pig. Muscle pH was measured using a pH meter from AZ Instrument Corp. (Taichung, Taiwan, China), calibrated with pH 7.00 and 4.00 buffer solution. Shear force of the LD was measured according to NY/T 821-2019 using a texture-measurement system equipped with a TMS load cell (Food Technology Corporation, Sterling, VA, USA). Sixteen amino acids were determined in acid-hydrolyzed LD samples using an UltiMate 3000 high-performance liquid chromatography system (Thermo Fisher Scientific, Waltham, MA, USA) equipped with an XSelect HSS T3 column (4.6 × 250 mm, 5 µm; Waters Corporation, Milford, MA, USA). The mobile phases were 10 mM KH_2_PO_4_ adjusted to pH 4.8 ± 0.1 (A) and methanol (B), with gradient elution and detection at 254 nm.

Liver samples were homogenized using a tissue homogenizer (Servicebio Technology, Wuhan, Hubei, China), and protein concentrations were determined using a BCA Protein Assay Kit (Beyotime Biotechnology, Shanghai, China) according to the manufacturer’s instructions. The BCA assay quantifies protein through the formation of a purple complex between bicinchoninic acid and cuprous ions generated by protein-mediated reduction in cupric ions under alkaline conditions [25]. Serum and hepatic total superoxide dismutase (T-SOD), catalase (CAT), glutathione peroxidase (GSH-Px), total antioxidant capacity (T-AOC), and malondialdehyde (MDA) were measured using assay kits from Nanjing Jiancheng Bioengineering Institute (Nanjing, Jiangsu, China) and a microplate reader (Millipore, Billerica, MA, USA). According to the supplied kit protocols, T-SOD was determined by the hydroxylamine method at 550 nm (A001-1 series); CAT by the ammonium molybdate method at 405 nm (A007-1-1); GSH-Px by the glutathione-consumption/DTNB method at 412 nm (A005-1 series); and MDA by the thiobarbituric acid method at 532 nm (A003-1 series). T-AOC was determined using the ABTS method with Trolox calibration at 405 nm (A015-2-1). GSH-Px denotes enzyme activity rather than glutathione concentration. Enzyme activities were expressed as U/mL in serum and U/mg protein in liver, and MDA as nmol/mL and nmol/mg protein, respectively. T-AOC was expressed as mmol/mL in serum and mmol/mg protein in liver.

### 2.3. Microbial DNA Extraction, Amplification and Community Analyses

Total genomic DNA was extracted from colonic content samples, and DNA integrity was evaluated by 1% agarose gel electrophoresis. The V3–V4 region of the bacterial 16S rRNA gene was amplified using the universal primer pair 338F (5′-ACTCCTACGGGAGGCAGCAG-3′) and 806R (5′-GGACTACHVGGGTWTCTAAT-3′), with sample-specific barcodes incorporated at the 5′ ends. PCR amplification was performed using TransStart FastPfu DNA Polymerase (TransGen, Beijing, China) on an ABI GeneAmp 9700 thermal cycler (Applied Biosystems, Foster City, CA, USA). Each sample was amplified in triplicate, and the replicate PCR products were pooled. Amplicons were examined by 2% agarose gel electrophoresis, purified using an AxyPrep DNA Gel Extraction Kit (Axygen Biosciences, Union City, CA, USA), eluted with Tris–HCl buffer, and quantified using a QuantiFluor-ST fluorescence system (Promega, Madison, WI, USA). Sequencing libraries were prepared using a TruSeq DNA Sample Preparation Kit (Illumina, San Diego, CA, USA) and subjected to paired-end sequencing on an Illumina platform at Majorbio Bio-Pharm Technology Co., Ltd., Shanghai, China. Raw paired-end reads were demultiplexed and quality-filtered using Fastp (0.19.6; HaploX, Shenzhen, Guangdong, China) and subsequently merged according to their overlapping regions. The resulting optimized sequences were denoised using the DADA2 plugin implemented in QIIME 2 (version 2024; QIIME2 Development Team, Flagstaff, AZ, USA) to generate amplicon sequence variants (ASVs). Taxonomic classification of representative ASV sequences was performed using the classify-sklearn naïve Bayes classifier in QIIME 2 against the SILVA 138.2/16s_bacteria reference database, with a confidence threshold of 70%. A total of 12 samples, comprising six samples per treatment, were included in the microbiota analyses. Chao1 richness and Shannon diversity indices were calculated to evaluate alpha diversity. Microbial community composition was visualized using principal coordinates analysis (PCoA) based on Bray–Curtis dissimilarities, as well as phylum- and genus-level relative-abundance profiles. PCoA was conducted at the ASV level, and differences in community structure between treatments were assessed using analysis of similarities (ANOSIM) with 999 permutations. Genus-level relative abundances were compared between the two treatments using the Wilcoxon rank-sum test in R (version 3.3.1; R Core Team, Vienna, Austria). Linear discriminant analysis effect size (LEfSe) was used to identify discriminative taxonomic features between the treatments [26]. Taxa with *p* < 0.05 and a linear discriminant analysis (LDA) score ≥ 2.5 were considered significantly discriminative. Some data analysis was performed using the Major Cloud Platform (Majorbio Bio-Pharm Technology Co., Ltd., Shanghai, China).

### 2.4. Statistical Analysis

Results are presented as the averages with standard error of the mean (SEM). Data were organized in Microsoft Excel (version 2016, Microsoft Corporation, Redmond, WA, USA). The original phenotypic analyses used independent-samples t-tests in IBM SPSS Statistics (version 25, IBM Corp., Armonk, NY, USA). Microbial comparisons and correlations are interpreted as exploratory. The allocation and sampling structure comprised three dietary replicate pens per treatment. For slaughter sampling, two pigs whose body weights were closest to the mean body weight of their respective pen were selected from each pen, resulting in six sampled pigs per treatment. The pen was considered the experimental unit for growth-performance variables (*n* = 3 pens per treatment). No formal a priori power analysis was performed to determine the number of replicate pens. For phenotypic analysis, statistical significance was set at *p* ≤ 0.05, with highly significant results at *p* < 0.01, and trends noted when 0.05 < *p* ≤ 0.1. For multiple genus-level comparisons, raw *p*-values were adjusted using the Benjamini–Hochberg false discovery rate (FDR) method, and FDR-adjusted *p* < 0.05 were considered statistically significant.

Associations between bacterial relative abundances and serum/hepatic antioxidant indices or muscle amino acid measurements were examined using correlation heatmaps. Relative-abundance data are compositional, and differential-abundance results can depend on the analytical method [27,28,29]. These associations were used to identify candidate relationships rather than to infer causal pathways.

## 3. Results

### 3.1. Growth Performance, Carcass Traits and Meat Quality

Using the pen as the experimental unit (*n* = 3 pens per treatment), no statistically significant between-treatment differences were detected in initial or final body weight, ADG, ADFI, or feed-to-gain ratio (all *p* > 0.05; Table 2). Final body weights were 133.15 and 134.74 kg in the control and TP groups, respectively. ADG was 982.50 versus 1005.86 g/day, ADFI was 3474.26 versus 3440.98 g/day, and feed-to-gain ratio was 3.53 versus 3.42 (all *p* > 0.05). Compared with Control group, TP-fed pigs had greater carcass weight (*p* = 0.024) and backfat thickness (*p* = 0.001; Table 3). Muscle lightness (L*: *p* = 0.011) and yellowness (b*: *p* = 0.028) were lower in the TP group. Drip loss, pH at 24 h, marbling score, meat color score, redness, myoglobin, shear force, moisture, IMP, and inosine did not differ significantly between treatments (all *p* > 0.05; Table 3). Muscle pH was 5.40 in Control group and 5.54 in TP-fed pigs, while drip loss was 2.77% and 2.14%, respectively. Muscle alanine was higher in the TP group than in Control group (*p* = 0.048; Table 4), representing a 17.5% increase calculated from the group means. The other 15 measured amino acids did not differ significantly between treatments (all *p* > 0.05). These measurements represent total amino acid concentrations determined after acid hydrolysis and therefore do not reflect the free, taste-active amino acid fraction.

### 3.2. Serum and Hepatic Antioxidant Indices

**Figure 1:**
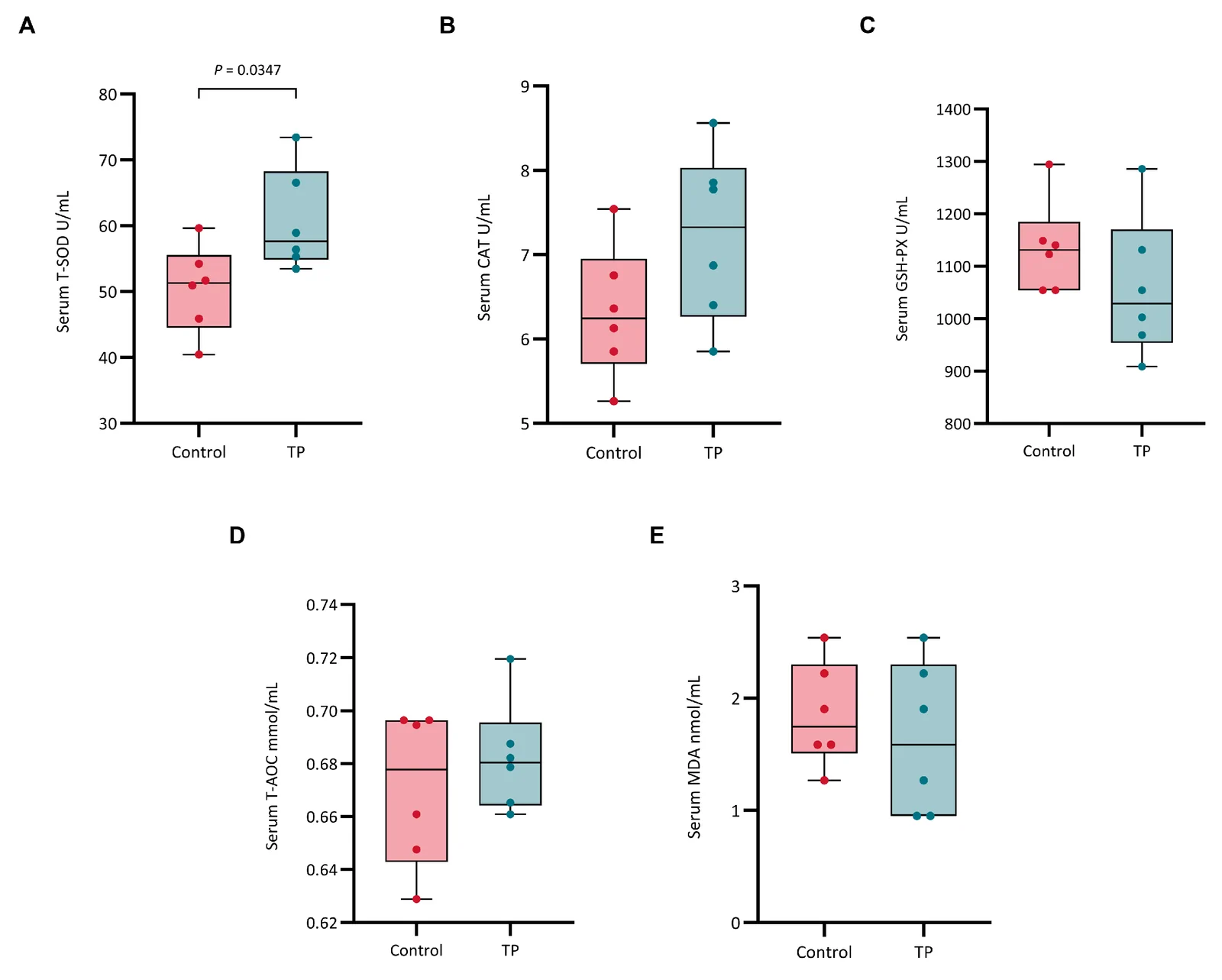
Serum antioxidant indices in control and TP-fed finishing pigs. (**A**) T-SOD; (**B**) CAT; (**C**) GSH-Px; (**D**) T-AOC; (**E**) MDA. Plots show individual points with box plots. Values are normalized to protein as shown on the source axes. T-AOC is expressed as mmol/mg protein (*n* = 6).

**Figure 2:**
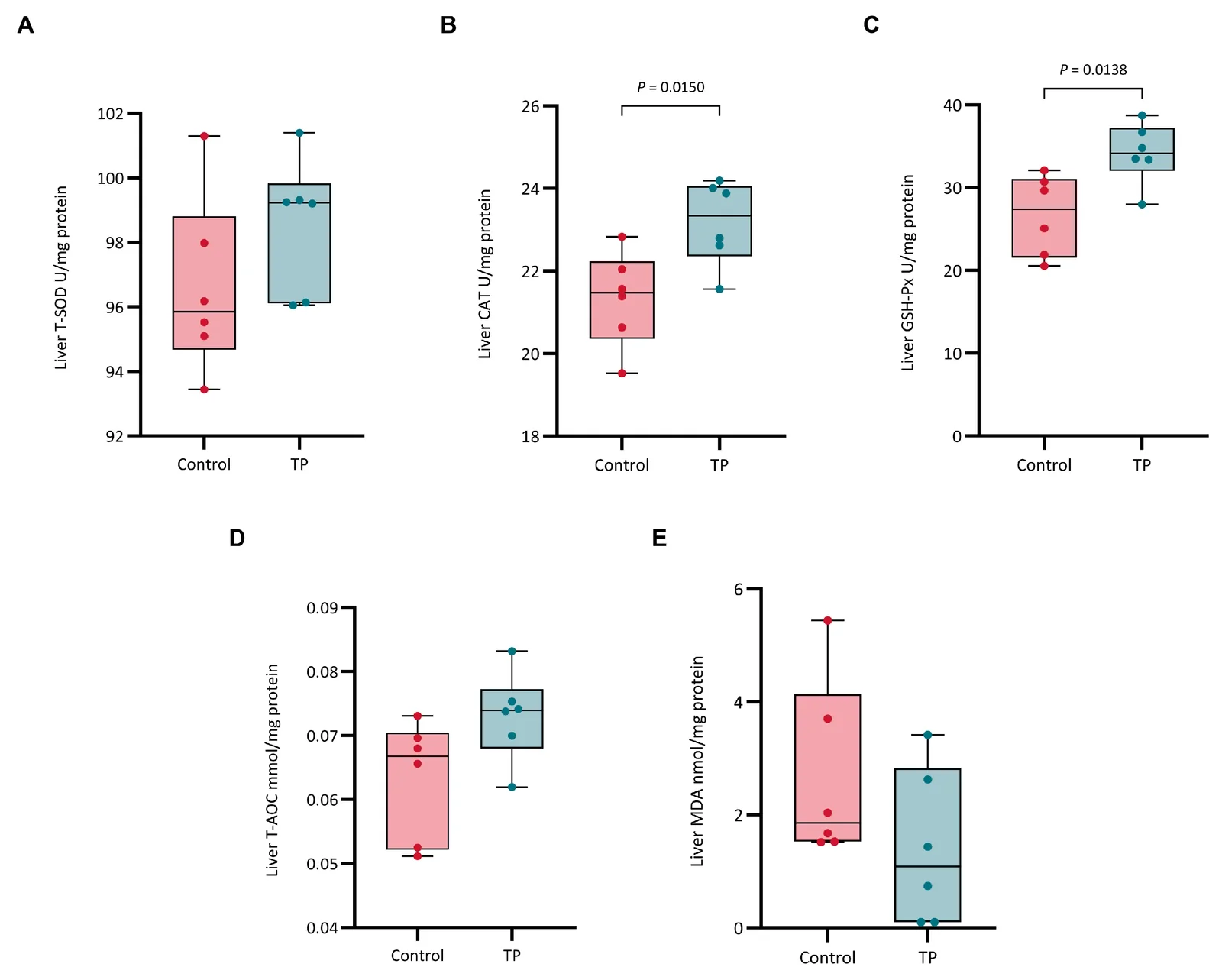
Hepatic antioxidant indices in control and TP-fed finishing pigs. (**A**) T-SOD; (**B**) CAT; (**C**) GSH-Px; (**D**) T-AOC; (**E**) MDA (*n* = 6).

Serum T-SOD activity was higher in TP-fed pigs than in Control group (*p* = 0.035; Figure 1A). Hepatic CAT (*p* = 0.015; Figure 2B) and GSH-Px activities (*p* = 0.014; Figure 2C) were also higher. Hepatic T-SOD and T-AOC were numerically higher and hepatic MDA was numerically lower in the TP group. The remaining panels carry no significance annotations and are described as numerical responses.

### 3.3. Colonic Microbial Diversity and Composition

**Figure 3:**
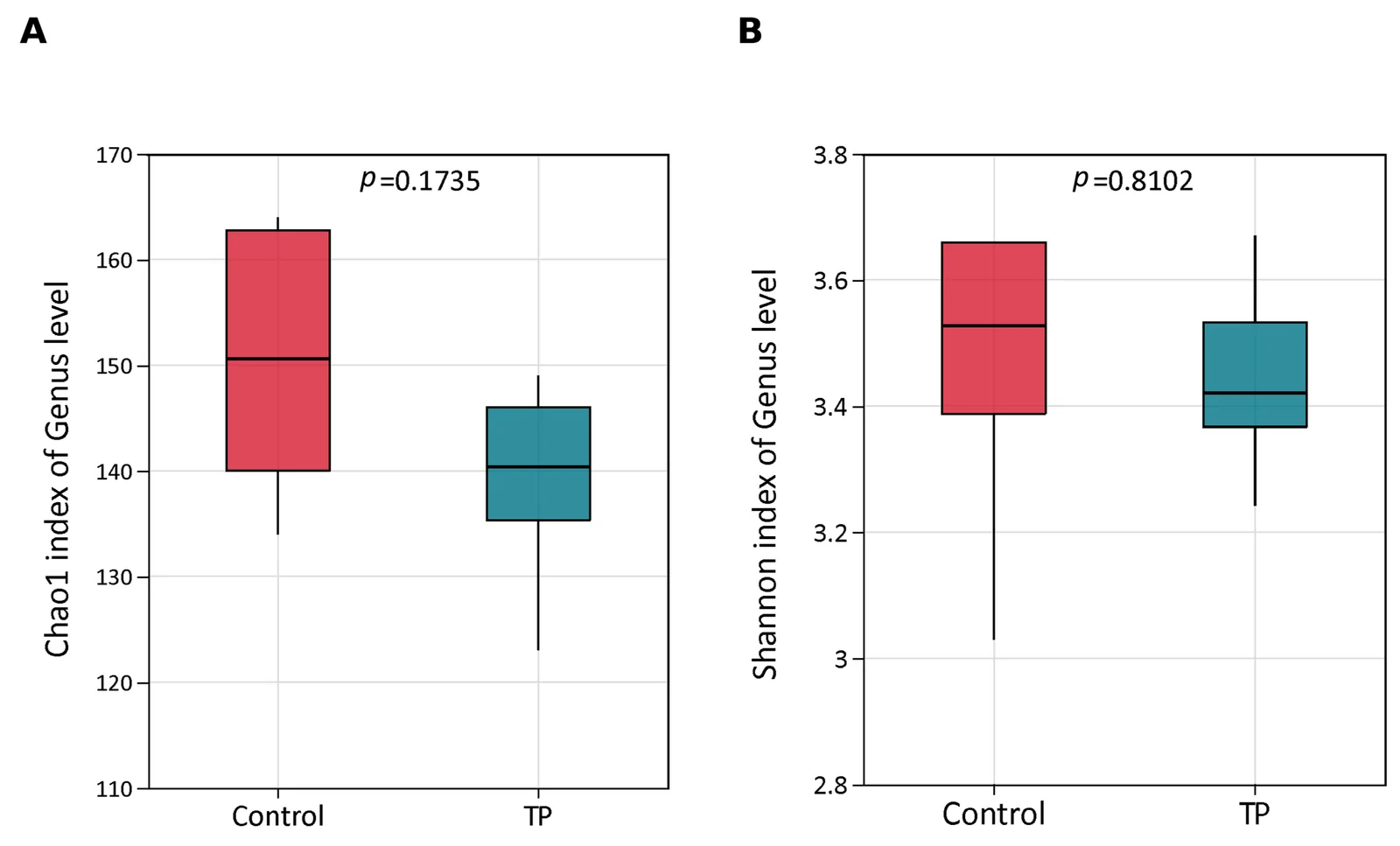
Alpha diversity of colonic microbial communities in control and TP groups. (**A**) Chao1 richness. (**B**) Shannon diversity (*n* = 6).

**Figure 4:**
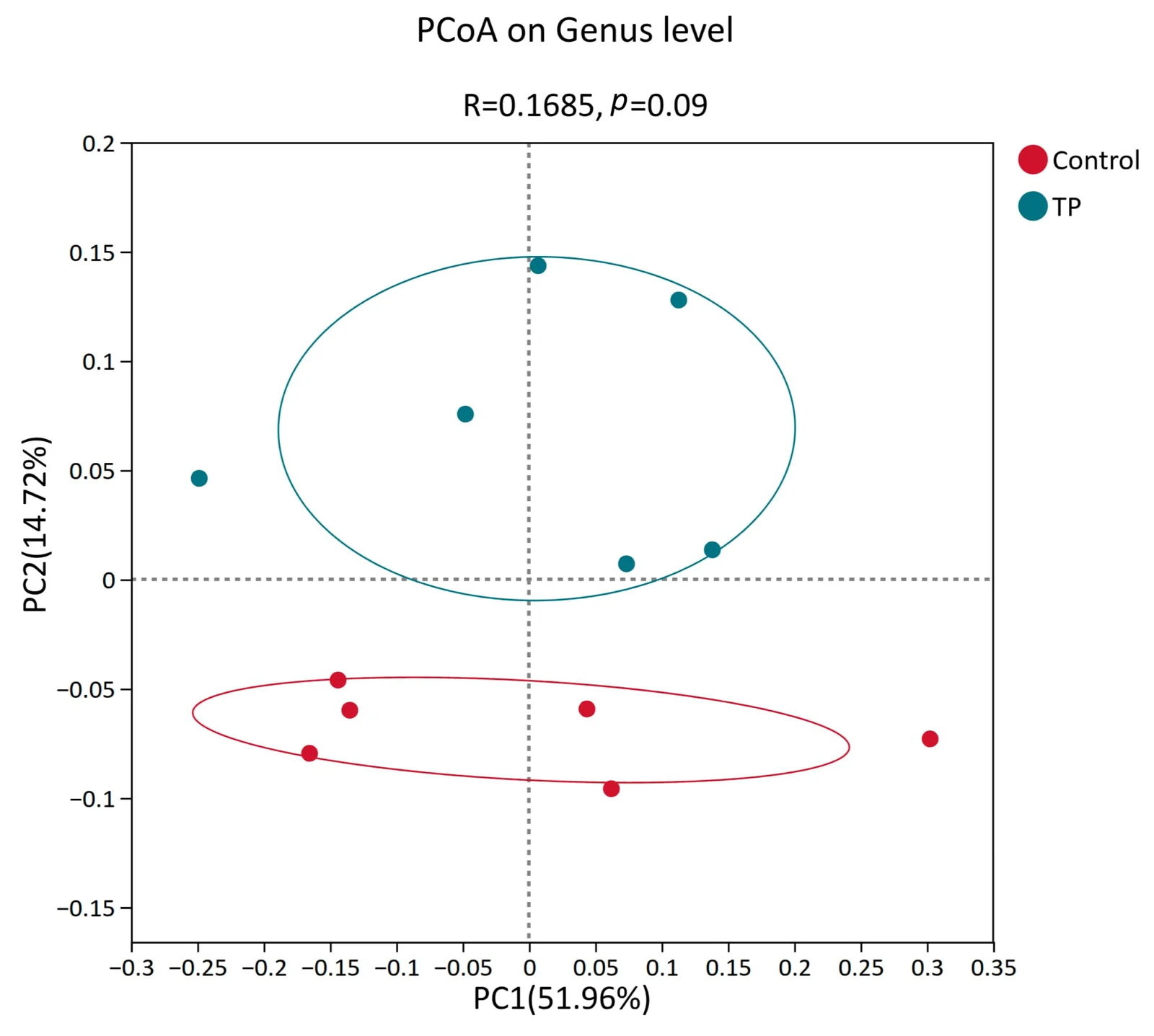
Bray–Curtis principal coordinates analysis of colonic microbial communities at genus level (*n* = 6). Gray dashed lines indicate the zero coordinates of PC1 and PC2 and are shown as visual reference lines.

**Figure 5:**
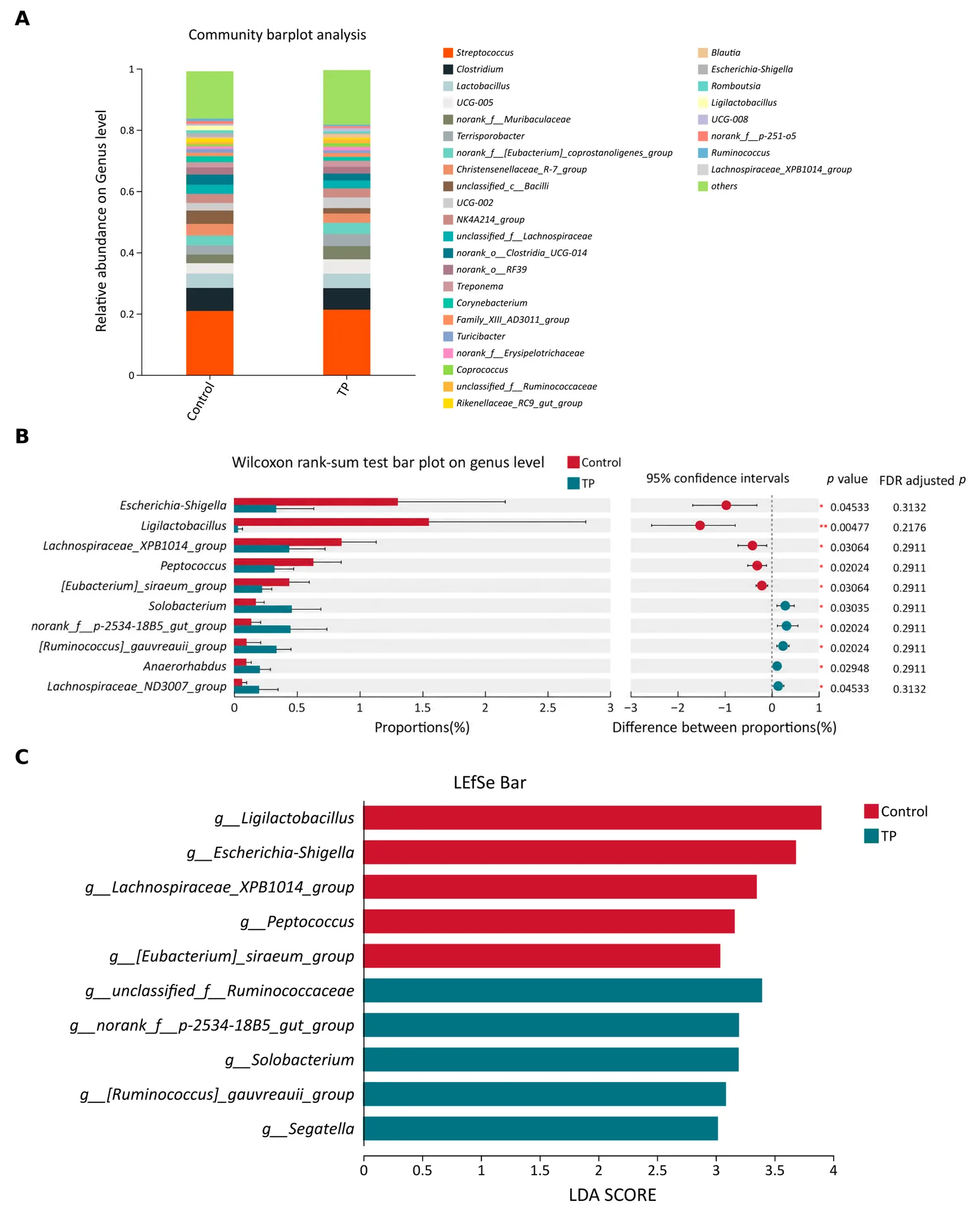
Colonic microbial composition and exploratory differential features on genus level. (**A**) Relative abundance profiles. (**B**) Wilcoxon rank-sum comparisons of selected genera. Intervals are labeled 95% confidence intervals in the source output. Both nominal *p*-values and false discovery rate (FDR)-adjusted *p*-values are presented. Asterisks indicate nominal significance (*p* < 0.05; *p* < 0.01); none of the comparisons remained significant after FDR correction. (**C**) LEfSe LDA bars, including genus- and species-level features (LDA score > 3.0).

As shown in Figure 3, Chao1 and Shannon indices did not differ significantly between the Control and TP groups (both *p* > 0.05, respectively), indicating that no statistically detectable between-group differences in microbial richness or within-sample diversity were observed. Genus-level PCoA showed partial separation between the two groups, with PC1 and PC2 explaining 51.96% and 14.72% of the variation, respectively (66.68% in total), but the difference in overall community structure did not reach statistical significance (*p* = 0.090; Figure 4). At the genus level, the microbial communities were predominantly composed of *Streptococcus*, *Clostridium*, *Lactobacillus*, *UCG-005*, *norank_f__Muribaculaceae*, and *Terrisporobacter*, among other taxa (Figure 5A). Based on the nominal, unadjusted *p*-values, Wilcoxon rank-sum tests showed that the relative abundances of *Escherichia–Shigella* (*p* = 0.0453), *Ligilactobacillus* (*p* = 0.0048), *Lachnospiraceae_XPB1014_group* (*p* = 0.0306), *Peptococcus* (*p* = 0.0202), and *[Eubacterium]_siraeum_group* (*p* = 0.0306) were lower in the TP group than in the Control group. In contrast, *Solobacterium* (*p* = 0.0304), *norank_f__p-2534-18B5_gut_group* (*p* = 0.0202), *[Ruminococcus]_gauvreauii_group* (*p* = 0.0202), *Anaerorhabdus* (*p* = 0.02948), and *Lachnospiraceae_ND3007_group* (*p* = 0.0453) were more abundant in the TP group (Figure 5B). However, none of these genus-level differences remained statistically significant after Benjamini–Hochberg FDR correction (all FDR-adjusted *p* > 0.05); therefore, these taxon-specific patterns were considered exploratory. Exploratory LEfSe analysis identified *Ligilactobacillus*, *Escherichia–Shigella*, *Lachnospiraceae_XPB1014_group*, *Peptococcus*, and *[Eubacterium]_siraeum_group* as Control-associated taxa, whereas *unclassified_f__Ruminococcaceae*, *norank_f__p-2534-18B5_gut_group*, *Solobacterium*, *[Ruminococcus]_gauvreauii_group*, and *Segatella* were associated with the TP group (Figure 5C). At the phylum level, Bacillota predominated in both groups, followed mainly by Bacteroidota and Actinomycetota (Figure S1A). The relative abundance of Pseudomonadota was nominally lower in the TP group than in the Control group (unadjusted *p* = 0.0453; Figure S1B). Collectively, TP supplementation was associated with exploratory differences in the relative abundances of selected bacterial taxa, whereas no statistically significant differences were detected in α-diversity or overall β-diversity.

### 3.4. Associations of Colonic Microbiota with Antioxidant Indices and Muscle Amino Acids

**Figure 6:**
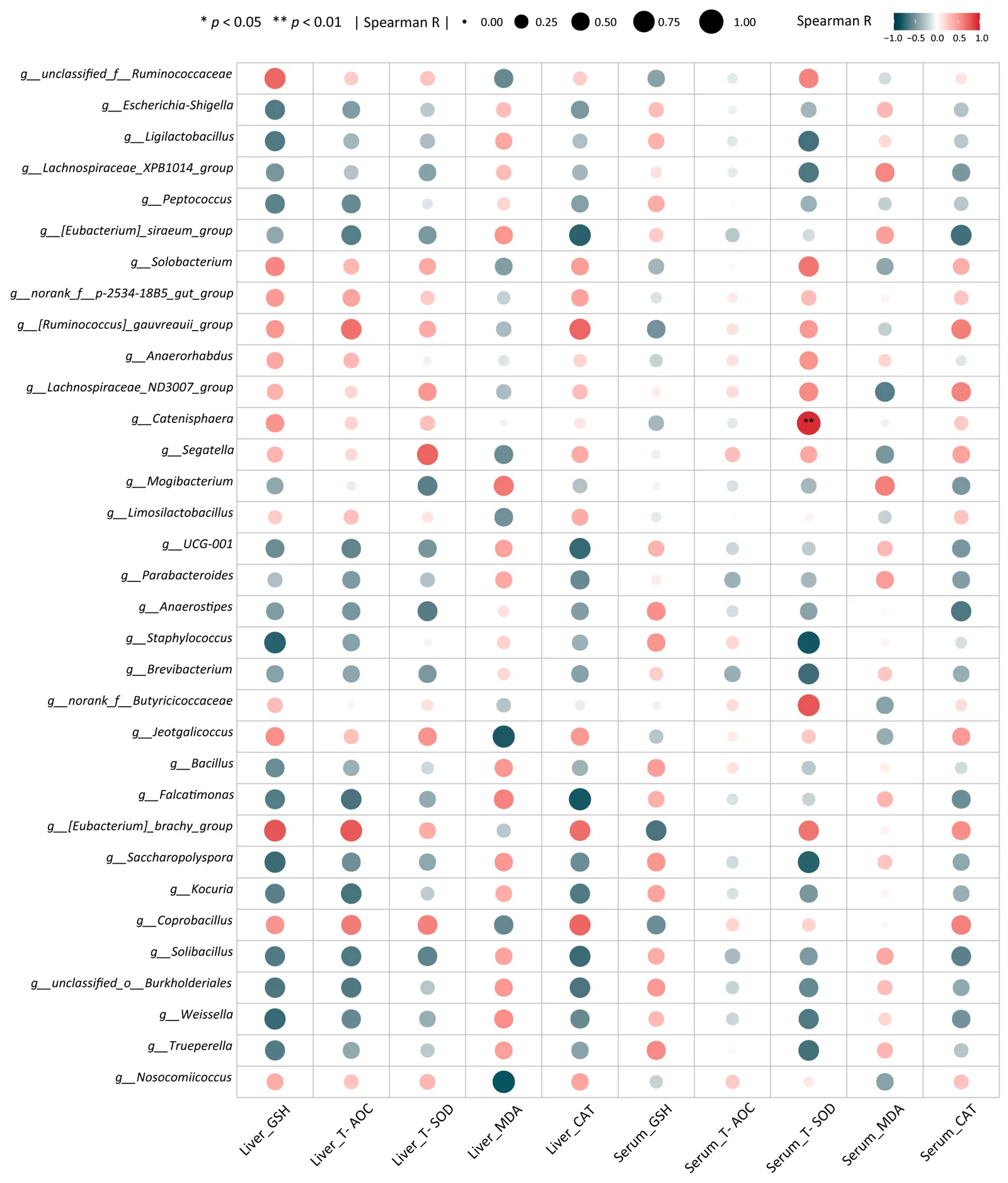
Spearman correlation analysis between the colonic bacterial community and serum and hepatic antioxidant indicators. Red and blue indicate positive and negative associations; symbol size reflects coefficient magnitude.

Spearman correlation analysis showed that *Catenisphaera* was positively correlated with serum total superoxide dismutase (T-SOD) activity (*p* < 0.01; Figure 6). Several additional, although statistically nonsignificant, correlation patterns were observed. *Ligilactobacillus* tended to be negatively associated with hepatic GSH, T-AOC, T-SOD, and CAT, as well as serum T-SOD, but positively associated with hepatic MDA. *Escherichia–Shigella* similarly showed negative correlation trends with several hepatic antioxidant indices, including GSH, T-AOC, T-SOD, and CAT. In contrast, *Solobacterium* and *Segatella* tended to be positively associated with hepatic T-SOD and CAT and negatively associated with hepatic MDA. *[Ruminococcus]_gauvreauii_group* also exhibited positive correlation trends with hepatic CAT, serum T-SOD, and serum CAT, together with negative trends with hepatic MDA and serum GSH. In addition, the analysis of muscle amino acids showed that *norank_f__p-2534-18B5_gut_group* was positively correlated with alanine content (*p* < 0.05; Figure S2). This taxon also exhibited generally positive correlation trends with several other muscle amino acids and was more abundant in the TP group in the Wilcoxon and LEfSe analyses (Figure 5B,C). By contrast, *Ligilactobacillus* and *Peptococcus* showed relatively strong but *nonsignificant* negative correlation trends with alanine and several other amino acids. *Limosilactobacillus* tended to be positively associated with multiple muscle amino acids, whereas *Parabacteroides* generally displayed negative correlation trends with several amino acids. *Solobacterium* showed negative trends with serine, glutamate, and glycine, while *Segatella* tended to be positively associated with alanine and methionine.

## 4. Discussion

### 4.1. Growth Performance and Meat Quality

Feed utilization efficiency, carcass composition, and meat quality reflect different aspects of the production value of finishing pigs. The feed-to-gain ratio measures the amount of feed consumed per unit of live weight gain [30], whereas backfat thickness reflects the degree of subcutaneous fat deposition; meat color and myoglobin content are related to muscle fiber characteristics [31]. Previous studies have generally suggested that tea polyphenols can improve growth performance and meat quality in finishing pigs. A study reported that dietary supplementation with 0.5–1% green tea by-products increased slaughter weight and meat shear force in finishing pigs [32]. Norkeaw et al. [33] found that dietary supplementation with 500 mg/kg green tea extract improved the oxidative stability of the *longissimus dorsi* during 6 d of chilled storage. In the present study, the concurrent increases in carcass weight and backfat thickness, despite the absence of significant changes in final body weight, ADG, or ADFI, may suggest altered nutrient partitioning toward subcutaneous fat deposition rather than a general enhancement of growth. Differences from previous findings may be related to the composition and dose of tea-derived products, pig genotype, and physiological stage. The biological mechanisms underlying the increased backfat thickness require further investigation through assessments of adipose tissue morphology and related metabolic indicators. ADFI and the feed-to-gain ratio were numerically lower in the TP group, although the differences were not statistically significant. Because the analysis included only three replicate pens per treatment and no a priori power analysis was performed, the study may have had limited power to detect modest treatment effects. These nonsignificant findings should therefore be interpreted cautiously and confirmed in studies with additional replicate pens. Regarding meat quality, the most evident change in meat color was lower L* and b* values at 24 h postmortem, indicating a darker and less yellow LD muscle. Pork color depends on pigment content and its redox state, postmortem metabolism, and light-scattering properties within the muscle [34]. Slow-oxidative type I muscle fibers and type IIa fibers with greater oxidative metabolic capacity differ metabolically from fast-glycolytic type IIb fibers. Ryu and Kim [35] found in pig *longissimus dorsi* that higher area proportions of type I and IIa fibers were associated with lower drip loss, whereas higher proportions of type IIb fibers were associated with faster postmortem metabolism and poorer water-holding capacity. For the TP group, myoglobin content and a* value were numerically higher. A possible link between these changes and muscle oxidative metabolic characteristics is biologically plausible.

Drip loss was numerically lower and moisture content was numerically higher, but neither differed significantly. The effects of other natural phenolic compounds also vary by endpoint: resveratrol supplementation altered muscle fiber characteristics and anti-oxidant status [36], while long-term supplementation increased intramuscular fat and altered lipid-related gene expression [37]. Interventions with oregano essential oil or quercetin, as well as another study on natural phenolic supplementation in finishing pigs, produced different combinations of antioxidant and meat quality responses [38,39]. Muscle total alanine content was significantly increased, whereas total glutamate and glycine were numerically higher but not significantly. Consistent with the possibility that carcass composition and amino acid availability can respond of overall growth, Deng et al. found that low-protein diet without soybean meal altered plasma free amino acid concentrations and increased backfat thickness without significantly affecting growth performances in finishing pigs [40]. These amino acids are relevant to flavor studies because free alanine and glycine contribute to sweetness, while free glutamate contributes to umami. Yang et al. [41] linked differences in taste-active metabolites to sensory attributes in processed pork hock products. However, the present measurements represent total amino acids determined after acid hydrolysis rather than free amino acid concentrations. Therefore, the increase in alanine should be interpreted as a change in the amino acid composition of the longissimus dorsi muscle rather than direct evidence of altered taste or flavor. Its potential sensory relevance should be further clarified by free amino acid profiling, cooking-related volatile compound analysis, and sensory evaluation.

### 4.2. Serum and Hepatic Antioxidant Indicators

Previous pig studies have indicated that tea polyphenols and tea-derived components have the potential to modulate systemic antioxidant defense. A study in finishing pig diets found that green tea extract containing linseed significantly improved the oxidative stability of blood and pork [42]. Zhang et al. [43] found that dietary supplementation with a mixture of tea polyphenols and sweetener increased the antioxidant capacity of serum and *longissimus dorsi* muscle in finishing pigs. These results have mostly focused on endpoints such as serum, plasma, or muscle, but evidence for synchronous responses in liver remains limited. In this study, increased serum T-SOD activity was observed, providing complementary evidence. In addition, hepatic CAT and GSH-Px activities were elevated, suggesting that tea polyphenol supplementation may enhance the liver’s capacity to defend against H_2_O_2_- and lipid peroxide-induced oxidative damage to proteins and DNA and to protect cell membrane integrity. Although not significant, the concurrent decrease in MDA and increase in T-AOC further suggest that tea polyphenols may improve systemic redox balance. Taken together, TP supplementation selectively enhanced enzymatic antioxidant defense, as evidenced by increased serum T-SOD activity and hepatic CAT and GSH-Px activities.

Host antioxidant responses to dietary TP may vary with the supplementation level and formulation. Supplementation with 0.4 g TP/kg diet was previously reported to increase T-SOD and GSH-Px activities in the serum and *longissimus dorsi* of finishing pigs [43], whereas approximately 0.3 g TP/kg diet in the present study increased serum T-SOD activity and hepatic CAT and GSH-Px activities. These findings suggest that selected enzymatic antioxidant responses may depend on the supplementation level and target tissue, while context- and exposure-dependent redox regulation has also been observed in microorganisms. However, enzyme activity is not equivalent to ROS levels or to the cumulative damage caused by ROS [44]. Moreover, different antioxidant assays have different chemical bases. The ABTS method reflects the ability to eliminate ABTS free radical cations, which is expressed in Trolox equivalents [45], and the FRAP method measures the Fe^3+^ reduction ability [46]. These two methods cannot replace endpoint indicators such as enzyme activity and MDA, so the indicators do not need to be consistent. Similar inconsistencies have also been observed in previous studies. Wang et al. [8] found that plasma glutathione concentration increased in piglets supplemented with tea residue, but the measured enzyme activities did not increase significantly. A study on natural phenols evaluated glutathione, enzyme activity, scavenging ability and lipid oxidation, respectively [39]. These discrepancies may be related to the dosage, pig growth stage, and tissue specificity. The tea polyphenols undergo absorption and first-pass metabolism in the liver, resulting in different blood and liver exposures as well as metabolic profiles, thereby contributing to tissue-specific response patterns. Future studies should further measure absorbed tea-derived metabolites to elucidate the molecular mechanisms by which tea polyphenols enhance antioxidant capacity in pigs.

### 4.3. Colonic Community Structure and Antioxidant-Associated Taxa

Tea components can both influence bacterial growth and be biotransformed by microorganisms [9,10,11,12]. Zhu et al. [47] reported that piceatannol enhanced antioxidant capacity and altered intestinal microbial composition in weaned piglets, with these changes being associated with antioxidant function. Sun et al. [48] reported that *Lactobacillus salivarius* alleviated intestinal inflammation and oxidative stress in lipopolysaccharide-challenged piglets. In this study, no statistically significant differences were detected in Chao1 richness or Shannon diversity, and the treatment-related difference in genus-level community structure did not reach statistical significance in the PCoA analysis (*p* = 0.090). The microbial response to TP may therefore involve exploratory changes in selected taxa rather than extensive remodeling of the overall colonic community structure. At the phylum level, Bacillota remained predominant in both groups, whereas the relative abundance of Pseudomonadota was nominally lower in the TP group. At the genus level, *Escherichia–Shigella* showed a nominally lower relative abundance in the TP group, which may indicate a potentially favorable ecological shift because this taxonomic group includes facultative anaerobic and potentially opportunistic bacteria. In weaned piglets, enterotoxigenic *Escherichia coli* K88 infection has been shown to promote intestinal injury and diarrhea through activation of the NLRP3 inflammasome–pyroptosis pathway [49]. However, this genus-level finding does not establish a reduction in pathogenic strains or inflammasome activity in the finishing pigs studied here. Meanwhile, TP supplementation increased *Solobacterium*, *norank_f__p-2534-18B5_gut_group*, *[Ruminococcus]_gauvreauii_group, Anaerorhabdus*, and *Lachnospiraceae_ND3007_group*, while LEfSe additionally identified *unclassified_f__Ruminococcaceae and Segatella* as TP-associated taxa. These findings suggest that TP primarily affected selected members of the colonic community. Collectively, these analyses identified candidate taxon-specific responses to TP supplementation that require validation in larger studies. Regarding the microbiota–amino acid relationship, *norank_f__p-2534-18B5_gut_group* was positively correlated with muscle alanine content and was consistently enriched in the TP group in both the Wilcoxon and LEfSe analyses. Previous pig studies provide indirect support for links between microorganisms and muscle metabolism and meat quality. Liu et al. [50] combined L-arginine with the microbiota and metabolic muscle profiles, and discovered changes in phospholipid metabolites, microbiota, meat color, and water loss. Very-low-protein diets altered the cecal microbiota and muscle amino acids and fatty acids [51]. Sebastià et al. [52] reported associations among rectal microbiota composition, short-chain fatty acids, and backfat and muscle fatty acid profiles, providing indirect evidence that the porcine gut microbiota may be associated with host tissue metabolic composition. For the genera identified in this study, Song et al. [53] discovered that *Mogibacterium* in the pig’s intestinal tract was negatively correlated with some intestinal cavity amino acids. Sabino de Oliveira et al. [54] found that *Acutalibacter* was associated with eQTLs in the brain and muscles of the host. These observations support further investigation of host–microbial metabolic relationships. Dietary amino acids and host metabolism can also inversely shape the microbiota [55]. Therefore, subsequent analysis of colon and circulating metabolites is helpful in distinguishing between microbial substrate utilization and changes in host nutritional availability, thereby further clarifying the causal relationships among the microbiota, amino acids, and antioxidant status in pigs after tea polyphenols treatment.

## 5. Conclusions

In summary, at the supplementation level evaluated in this study (300 mg TP/kg diet), no statistically detectable differences in growth performance were observed, although feed intake and feed-to-gain ratio were numerically lower; carcass weight, backfat thickness, and muscle alanine increased, while LD lightness and yellowness decreased. At the microbial level, TP supplementation did not significantly alter Chao1 richness, Shannon diversity, or overall colonic community structure, but selectively changed the relative abundances of specific bacterial taxa, including reductions in *Pseudomonadota* and *Escherichia–Shigella* and enrichment of several TP-associated taxa, such as *Solobacterium*, *norank_f__p-2534-18B5_gut_group*, *[Ruminococcus]_gauvreauii_group*, and Segatella. Exploratory correlation analysis further showed that *Catenisphaera* was positively correlated with serum T-SOD activity, whereas the TP-enriched *norank_f__p-2534-18B5_gut_group* was positively correlated with muscle alanine content. These findings should be regarded as hypothesis-generating candidate associations rather than evidence of causality. By assessing serum, liver, muscle, and colonic microbiota in the same feeding experiment, this study supports mechanistic investigation of a potential gut–liver–muscle association. These findings also support the potential application of tea polyphenols in finishing-pig nutrition.

## Figures and Tables

**Table 1:** Composition and Nutrient Levels of the Basal Diets.

Ingredient or Nutrient	Finisher Diet 1 ^(1)^	Finisher Diet 2 ^(1)^
Corn, %	39.6	38.8
Soybean meal, %	14.7	14
Barley, %	20	20
Wheat bran, %	16	18
Soybean oil, %	1.5	1
Soybean hulls, %	4	4
Lysine, %	0.2	0.2
Premix ^(2)^, %	4	4
Total, %	100	100
Nutrient Levels ^(3)^		
DE, Kcal/kg	3091.51	3035.35
CP, %	14.53	13.67
CF, %	5.64	5.76
Calcium, %	0.85	0.85
Phosphorus, %	0.58	0.54
Lysine, %	0.86	0.75
Tryptophan, %	0.17	0.17

^(1)^ The control group received the basal diet, while the TP group received the basal diet supplemented with 2000 mg/kg tea extract containing at least 15% tea polyphenols. ^(2)^ The premix provided: vitamin A1, 1250 IU; vitamin D3, 2475 IU; vitamin E, 45 IU; vitamin K3, 2.7 mg; vitamin B1, 5.2 mg; vitamin B2, 15 mg; vitamin B6, 8.1 mg; vitamin B12, 4.5 mg; pantothenic acid, 42 mg; niacin, 52 mg; folic acid, 3.3 mg; biotin, 0.3 mg; Zn as ZnSO_4_·7H_2_O, 120 mg; Fe as FeSO_4_·7H_2_O, 175 mg; Cu as CuSO_4_·5H_2_O, 130 mg; Mn as MnSO_4_·H_2_O, 60 mg; Se as Na_2_SeO_3_, 0.3 mg per kilogram of complete feed. The total dietary copper concentration in the complete feed did not exceed the applicable regulatory limit. ^(3)^ Abbreviations: DE = digestible energy; CP = crude protein; CF = crude fiber. Nutrients levels were calculated on as-fed basis. Digestible energy and amino acid contents were calculated while other values were measured.

**Table 2:** Growth Performance of Finishing Pigs.

Item	Control	TP	*p*-Value
Average initial body weight (kg)	74.20 ± 1.53	74.38 ± 1.46	0.934
Average final body weight (kg)	133.15 ± 1.58	134.74 ± 1.92	0.537
Average daily gain (g/day)	982.50 ± 28.96	1005.86 ± 54.81	0.717
Average daily feed intake (g/day)	3474.26 ± 35.45	3440.98 ± 94.28	0.752
Feed-to-gain ratio (F/G)	3.53 ± 0.07	3.42 ± 0.09	0.359

Values are presented as mean ± SEM based on pen means. The pen was considered the experimental unit (*n* = 3 pens per treatment and 20 pigs per pen.) No pigs were removed before the end of experiment. Exact *p*-values for the treatment effect are shown in the final column.

**Table 3:** Meat Quality Traits of Finishing Pigs.

Item	Control	TP	*p*-Value
Backfat thickness (mm)	19.25 ± 0.98	24.97 ± 0.86	0.001
Carcass weight (kg)	95.82 ± 1.41	100.18 ± 0.84	0.024
Drip loss (%)	2.77 ± 0.29	2.14 ± 0.28	0.146
pH _24 h_	5.40 ± 0.02	5.54 ± 0.07	0.067
Marbling score	1.83 ± 0.31	1.50 ± 0.22	0.401
Meat color score	1.50 ± 0.22	2.00 ± 0.37	0.270
Lightness (L*)	51.44 ± 0.82	47.06 ± 1.15	0.011
Redness (a*)	10.18 ± 0.23	11.37 ± 0.87	0.234
Yellowness (b*)	13.85 ± 0.76	11.51 ± 0.77	0.028
Myoglobin (mg/g)	2.24 ± 0.05	2.26 ± 0.05	0.757
Shear force (N)	110.99 ± 12.44	124.40 ± 2.76	0.337
Water (%)	71.12 ± 1.23	72.94 ± 0.47	0.196
IMP (mg/g)	1.94 ± 0.07	2.10 ± 0.06	0.101
Inosine (mg/g)	0.29 ± 0.03	0.31 ± 0.02	0.728

Abbreviations: IMP = Inosine monophosphate. Values were presented as mean ± SEM. (*n* = 6). Exact *p*-values for the treatment effect are shown in the final column. The same data presentation and statistical notation are used in all subsequent tables.

**Table 4:** Amino Acid Composition of *Longissimus Dorsi* in Finishing Pigs.

Item	Control	TP	*p*-Value
Aspartic acid (Asp)	1.61 ± 0.11	1.86 ± 0.04	0.085
Threonine (Thr)	0.83 ± 0.05	0.86 ± 0.02	0.523
Serine (Ser)	0.71 ± 0.05	0.71 ± 0.03	0.924
Glutamic acid (Glu)	2.86 ± 0.17	3.12 ± 0.10	0.217
Glycine (Gly)	0.82 ± 0.04	0.85 ± 0.01	0.448
Alanine (Ala)	1.03 ± 0.07	1.21 ± 0.03	0.048
Valine (Val)	0.91 ± 0.08	1.04 ± 0.03	0.137
Methionine (Met)	0.43 ± 0.03	0.49 ± 0.02	0.128
Isoleucine (Ile)	0.86 ± 0.06	1.00 ± 0.06	0.122
Leucine (Leu)	1.53 ± 0.09	1.62 ± 0.04	0.401
Tyrosine (Tyr)	0.58 ± 0.04	0.61 ± 0.02	0.468
Phenylalanine (Phe)	0.75 ± 0.05	0.80 ± 0.02	0.300
Lysine (Lys)	1.64 ± 0.11	1.76 ± 0.03	0.354
Histidine (His)	0.89 ± 0.07	0.94 ± 0.02	0.442
Arginine (Arg)	1.16 ± 0.07	1.23 ± 0.02	0.370
Proline (Pro)	0.73 ± 0.06	0.78 ± 0.02	0.435

## Data Availability

The original contributions presented in this study are included in the article. This data can be found in the SRA database (https://www.ncbi.nlm.nih.gov/bioproject/PRJNA1529872, accessed on 6 October 2026).

## Supplementary Materials

The following supporting information can be downloaded at: https://www.mdpi.com/article/10.3390/ani16193135/s1. Figure S1: Colonic microbial composition and exploratory differential features on phylum level. Figure S2: Spearman correlation analysis between the colonic bacterial community and muscle amino acids.
