# Supplementary material for: Dietary Tea Polyphenols Improve Meat Quality of Finishing Pigs by Enhancing Antioxidant Enzyme Activities and Modulating Gut Microbiota
Source: Animals (Basel). 2026 Oct 7;16(19):3135. doi: 10.3390/ani16193135 (PMC13663568; doi:10.3390/ani16193135)
Supplement: Supplementary file 1 [file animals-16-03135-s001.zip › animals-4599678-supplementary.pdf]

Supplementary Materials

Dietary Tea Polyphenols Improve Meat Quality of Finishing Pigs by Enhancing Antioxidant Enzyme Activities and Modulating Gut Microbiota

Zhixin Yu [1,2,3], Luhao Tian [1,2,3], Yanzhe Zhang [1,2,3], Haoneng Guo [1,2,3], Zhixuan Zhang [1,2,3], Lexuan Shi [1,2,3], Zengzhuang Yuan [4], Zeqing Lu [1,2,3], Yizhen Wang [1,2,3] and Yuanzhi Cheng [1,2,3,*]

[1] Zhejiang Key Laboratory of Nutrition and Breeding for High-Quality Animal Products, Zhejiang University, Hangzhou 310058, China; 12517064@zju.edu.cn (Z.Y. Zhixin Yu); 22517017@zju.edu.cn (L.T.); 22317020@zju.edu.cn (Y.Z.); 12517062@zju.edu.cn (H.G.); 22417014@zju.edu.cn (Z.Z.); 12417034@zju.edu.cn (L.S.); zqlu2012@zju.edu.cn (Z.L.); yzwang321@zju.edu.cn (Y.W.); yzcheng@zju.edu.cn (Y.C.)

[2] Key Laboratory of Molecular Animal Nutrition, Ministry of Education, Hangzhou 310058, China; 12517064@zju.edu.cn (Z.Y.); 22517017@zju.edu.cn (L.T.); 22317020@zju.edu.cn (Y.Z.); 12517062@zju.edu.cn (H.G.); 22417014@zju.edu.cn (Z.Z.); 12417034@zju.edu.cn (L.S.); zqlu2012@zju.edu.cn (Z.L.); yzwang321@zju.edu.cn (Y.W.); yzcheng@zju.edu.cn (Y.C.)

[3] Key Laboratory of Animal Nutrition and Feed Science (Eastern of China), Ministry of Agriculture and Rural Affairs, Hangzhou 310058, China; 12517064@zju.edu.cn (Z.Y.); 22517017@zju.edu.cn (L.T.); 22317020@zju.edu.cn (Y.Z.); 12517062@zju.edu.cn (H.G.); 22417014@zju.edu.cn (Z.Z.); 12417034@zju.edu.cn (L.S.); zqlu2012@zju.edu.cn (Z.L.); yzwang321@zju.edu.cn (Y.W.); yzcheng@zju.edu.cn (Y.C.)

[4] Bureau of Animal Husbandry and Veterinary, Jiaxing 314050 , China; yuanzhengzhuang@126.com (Z.Y. Zengzhuang Yuan)

* Correspondence: yzcheng@zju.edu.cn (Y.C.)

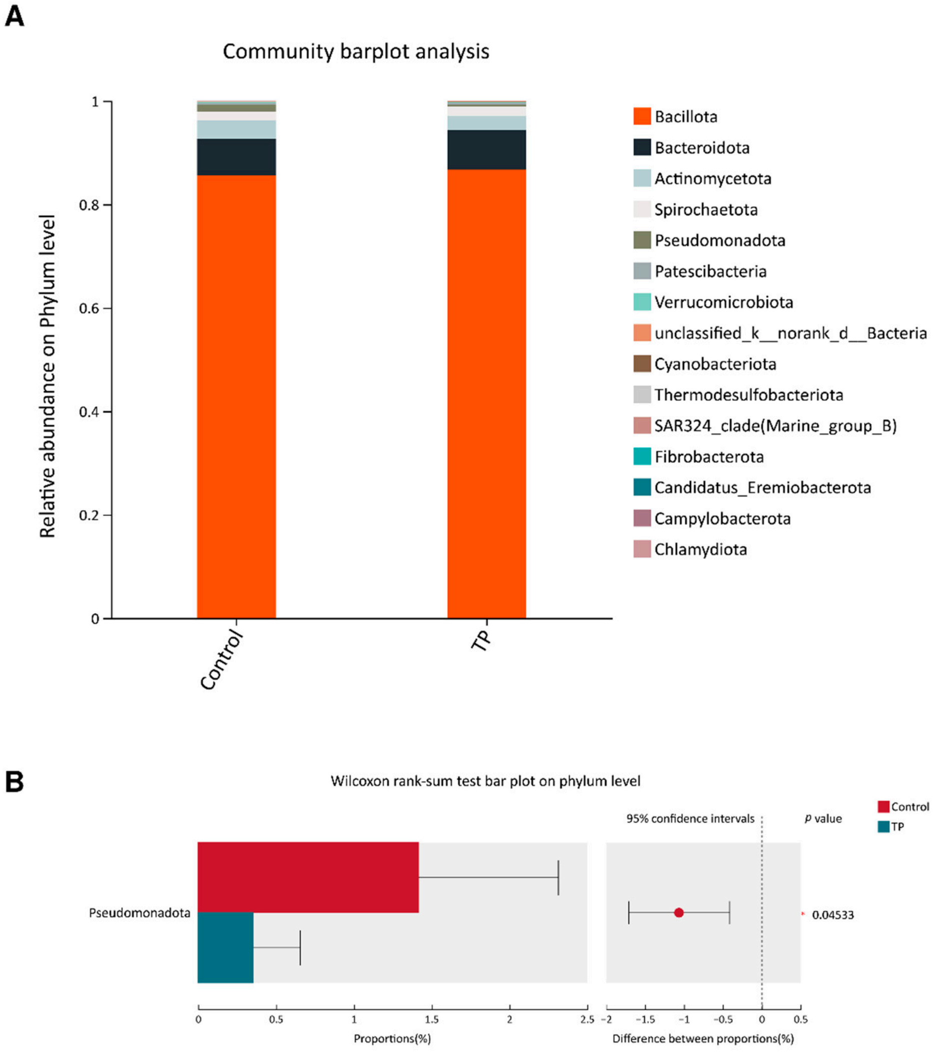

Figure S1. Colonic microbial composition and exploratory differential features on phylum level. (A) Relative-abundance profiles. (B) Wilcoxon rank-sum comparisons of selected genera. Intervals are labeled 95% confidence intervals in the source output, and p-values are nominal.

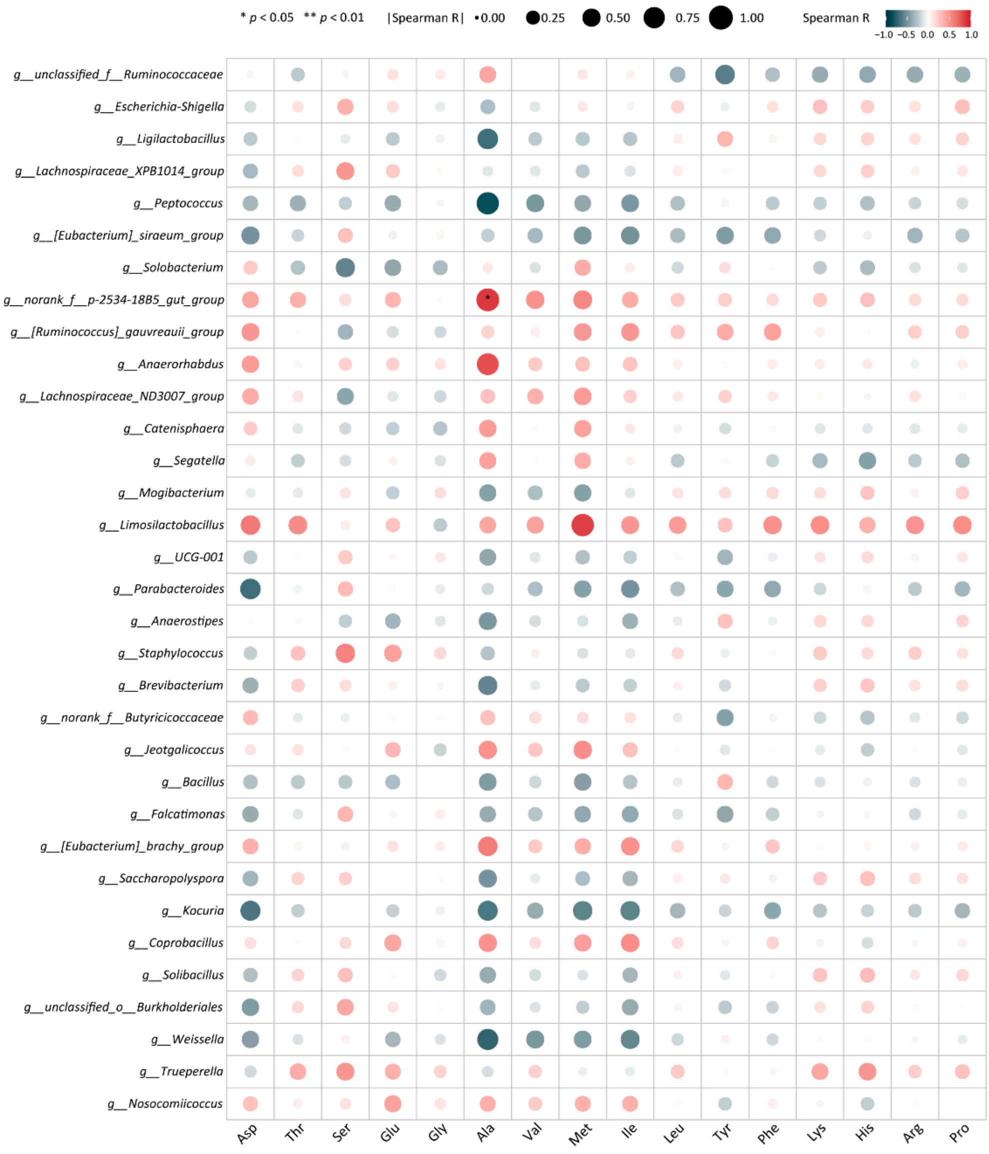

Figure S2. Spearman correlation analysis between the colonic bacterial community and muscle amino acids. Red and blue indicate positive and negative associations; symbol size reflects coefficient magnitude.
